# CCR9 Antagonists in the Treatment of Ulcerative Colitis

**DOI:** 10.1155/2015/628340

**Published:** 2015-09-20

**Authors:** Pirow Bekker, Karen Ebsworth, Matthew J. Walters, Robert D. Berahovich, Linda S. Ertl, Trevor T. Charvat, Sreenivas Punna, Jay P. Powers, James J. Campbell, Timothy J. Sullivan, Juan C. Jaen, Thomas J. Schall

**Affiliations:** ChemoCentryx, Inc., 850 Maude Avenue, Mountain View, CA 94043, USA

## Abstract

While it has long been established that the chemokine receptor CCR9 and its ligand CCL25 are essential for the movement of leukocytes into the small intestine and the development of small-intestinal inflammation, the role of this chemokine-receptor pair in colonic inflammation is not clear. Toward this end, we compared colonic CCL25 protein levels in healthy individuals to those in patients with ulcerative colitis. In addition, we determined the effect of CCR9 pharmacological inhibition in the *mdr1a* ^−/−^ mouse model of ulcerative colitis. Colon samples from patients with ulcerative colitis had significantly higher levels of CCL25 protein compared to healthy controls, a finding mirrored in the *mdr1a* ^−/−^ mice. In the *mdr1a* ^−/−^ mice, CCR9 antagonists significantly decreased the extent of wasting and colonic remodeling and reduced the levels of inflammatory cytokines in the colon. These findings indicate that the CCR9:CCL25 pair plays a causative role in ulcerative colitis and suggest that CCR9 antagonists will provide a therapeutic benefit in patients with colonic inflammation.

## 1. Introduction

The chemokine system is an intricate network of related cytokines and their cognate receptors, which together play a key role in the directed migration of leukocytes to their site of action [1, 2]. Expression of both the chemokine receptor CCR9 and the integrin *α*_4_ *β*_7_ is required for efficient lymphocyte homing to the intestine; these proteins form the so-called gut-homing phenotype of intestinal lymphocytes [3, 4]. CCR9 is expressed on the majority of CD4^+^ (67%) and CD8^+^ (54%) T cells [5] as well as B cells [4] and plasmacytoid dendritic cells [6, 7] isolated from the small intestine. In contrast, 2–4% of circulating T cells in healthy individuals express CCR9 [5]. The only known ligand for CCR9, CCL25/TECK, is abundantly produced in the small intestine [8], where its expression is localized to the epithelium [5, 9, 10]. The requirement for CCL25 in the recruitment of lymphocytes to the intestine has been highlighted in CCL25-deficient mice, which display a lack of antigen-specific T cell recruitment into the lamina propria and epithelium [11]. Indeed, targeting the CCR9/CCL25 axis has been shown to be of benefit in the TNF-ΔARE [12] and SAMP-1/YIT [13] models of ileitis. These observations are also consistent with those made in an adoptive transfer model in which pharmacological inhibition of CCR9 resulted in a significant reduction in the numbers of T cells recruited in the small intestine, without inhibiting cell trafficking to other organs [14]. Indeed, in the PROTECT-1 clinical trial, pharmacological inhibition of CCR9 resulted in a significant reduction in disease severity in Crohn's disease subjects, relative to those on placebo [15].

While it is clear that CCL25 is produced in the small intestine and regulates migration of circulating CCR9^+^ leukocytes to this tissue under both homeostatic and inflammatory conditions, the role of CCR9 and CCL25 in leukocyte migration to the colon is controversial. Although CCR9^+^ cells have been found in normal human colon [5], until recently CCL25 had not [5, 9, 16]. In colon from inflammatory bowel disease (IBD) patients with active colonic inflammation, CCL25 mRNA was detected by one group [17] but not by two others [16, 18]. CCL25 mRNA was recently detected in mouse colon and was found to be upregulated in the DSS model of acute colitis [19]. An early report indicated that the increased frequency of circulating CCR9^+^ T cells observed in Crohn's disease patients was not observed if the inflammation was limited solely to the colon [18]. However, two recent reports have noted changes in circulating CCR9^+^ cells in individuals with purely colonic inflammation. Mann et al. reported that the percentages of circulating CCR9^+^ *γδ* T cells are increased in ulcerative colitis patients compared to healthy controls [20]. Linton et al. reported that CCR9 levels are higher on circulating T cells and HLA-DR^hi^ monocytes in IBD patients with active colonic inflammation compared to healthy controls [17]. These changes are reminiscent of those noted in patients with Crohn's disease [18] and are implicated in the pathogenesis of primary sclerosing cholangitis [21], an inflammatory liver disease with a high degree of association with ulcerative colitis [22, 23]. Importantly, the CCR9 antagonist Vercirnon/CCX282 reduced colonic inflammation in Crohn's disease patients with colonic involvement [15].

In this study, we investigated the role of CCR9 and CCL25 in colonic inflammation. First we demonstrate that CCL25 is expressed in healthy human colon and is localized to the epithelium. Then we show that the levels of colonic CCL25 protein are elevated, compared to healthy controls, both in patients with ulcerative colitis and in the* mdr1a* ^−*/*−^ mouse model of ulcerative colitis. Finally we show that two CCR9 small-molecule antagonists, both of which have been tested in humans [15], inhibit colonic inflammation in* mdr1a* ^−*/*−^ mice.

## 2. Materials and Methods

The selective small molecule CCR9 antagonists, CCX025 and CCX282-B, have been previously described [12, 24–26].

### 2.1. Northern Blotting

CCL25 and CCR9 DNA fragments were cloned from human thymus cDNA (Biochain, Hayward, CA) using primers 5′-ATGAACCTGTGGCTCCTG-3′ and 5′-TAACAGGCAGGAATGACTC-3′ (CCL25) and 5′-ATGACACCCACAGACTTC-3′ and 5′-GAGGGAGAGTGCTCCTG-3′ (CCR9). The fragments were isolated by agarose gel electrophoresis, labeled with ^32^P-cytidine 5′-triphosphate (GE Healthcare, Piscataway, NJ) using the NEBlot kit (New England Biolabs, Ipswich, MA) and purified on Sephadex G-50 columns (GE Healthcare, Piscataway, NJ). Normal tissue mRNAs were purchased from Ambion (Austin, TX), Biochain (Hayward, CA), Origene (Rockville, MD), and Cybrdi (Frederick, MD). RNAs were subjected to agarose gel electrophoresis (2 *µ*g/lane) and blotted to nylon membranes using the Northernmax kit (Ambion, Austin, TX). The membranes were prehybridized with Ultrahyb (Ambion, Austin, TX) at 42°C for 1 hr, and the radiolabeled probes were boiled and added (~5 × 10^6^ cpm/blot). After overnight hybridization at 42°C, the probes were removed and the blots were rinsed successively with 2x SSC 0.1% SDS and 0.1x SSC 0.1% SDS at 42°C. Blots were exposed to Hyperfilm MP (GE Healthcare, Piscataway, NJ) which were developed with a Mini-Med 90 (AFP, Elmsford, NY).

### 2.2. Immunofluorescence and Immunohistochemistry

Normal small intestine and colon frozen sections were purchased from Biochain (Hayward, CA). Sections were fixed for 5 min in cold acetone, air-dried, rinsed for 5 min in TBS, and immersed for 30 min in TBS containing 5% goat serum, 1% BSA, 0.1% triton X-100, and 0.05% Tween-20. Sections were exposed to anti-CCL25/TECK or its IgG2b isotype Ab (both from R&D Systems, Minneapolis, MN) at 2.5 *µ*g/mL for 1 hr. Sections were rinsed for 3 × 2 min with TBST and exposed to Alexa Fluor 488-conjugated goat anti-mouse IgG (Jackson Immunoresearch, West Grove, PA) at 4 *µ*g/mL for 30 min. Sections were rinsed with PBS for 5 min and coverslips were mounted with Vectashield with DAPI (Vector Labs, Burlingame CA). For immunohistochemistry (IHC), the sections were treated as described above but were additionally pretreated to remove endogenous alkaline phosphatase (Dako, Carpinteria, CA) and biotin (Vector Labs, Burlingame, CA). CCL25 mAb was detected with biotinylated goat anti-mouse IgG (Jackson Immunoresearch, West Grove PA) at 1 : 1000 for 30 min. After rinsing with TBST, the sections were exposed to avidin-conjugated alkaline phosphatase (Dako, Carpinteria, CA) for 20 min. The sections were rinsed in TBST, stained with fuchsin+ reagent (Dako, Carpinteria, CA), rinsed in water for 2 min, counterstained with Mayer's hematoxylin (Sigma, St. Louis, MO) for 2 min, and rinsed in tap water for 3 min. Coverslips were mounted with Vectamount (Vector Labs, Burlingame, CA).

### 2.3. Tissue Cytokine Levels

Human terminal ileum and colon biopsies were obtained at baseline from Crohn's disease patients with a Crohn's Disease Activity Index (CDAI) ≥ 250 (PROTECT-1 trial) [15]. Since this was a multinational, multicenter clinical trial, central ethics committees reviewed the protocol and informed consent form and provided written approval prior to study initiation. All patients who agreed to undergo colonoscopic biopsies provided written informed consent prior to the biopsy procedure. Colon biopsies from healthy individuals were obtained from ILSbio (Chestertown, MD). Samples were homogenized on ice and homogenates were analyzed for CCL25 by ELISA (R&D Systems, Minneapolis, MN). Murine intestine tissue was harvested, cleaned, and snap-frozen. Homogenates were analyzed for CCL25, IL-1*β*, IL-6, IL-10, and IFN*γ* by ELISA (R&D Systems, Minneapolis, MN). Cytokine levels were normalized to total protein in the sample as determined by Bradford Reagent (Bio-Rad, Hercules, CA).

### 2.4. *mdr1a* ^−/−^ Mouse Model of Ulcerative Colitis

All animal experiments and procedures were approved by the ChemoCentryx Institutional Animal Care and Use Committee under protocol number CCX-154-2002. Female* mdr1a* ^−/−^ and wild-type FVB mice were purchased from Taconic (Germantown, NY). CCX025, formulated in 1% hydroxypropyl methylcellulose, was dosed at 100 mg/kg s.c. once daily. CCX282-B, formulated in 5% Cremophor, was dosed at 50 mg/kg c.c. twice daily. During the course of the study, body weights and the incidence of diarrhea were recorded on a weekly basis. Any animals that exhibited weight loss of greater than 20% of their peak body weight were euthanized. Final body weights for any euthanized or dead animals were carried forward for data analysis. Diarrhea was scored on a 0–5 scale; when animals reached a score of ≥3, their diarrhea was constant and irreversible and was thus considered as* established *diarrhea.

### 2.5. Histological Analysis

Histological analysis of H&E stained samples was conducted in a blinded fashion by a board-certified veterinary pathologist. Extent of leukocyte infiltration, reported as inflammation score, was defined as follows: 0: normal; 1: minimal—diffuse minimal with no separation of glands; 2: mild—diffuse mild with no separation of glands; 3: moderate—diffuse moderate with mild multifocal separation of glands; 4: marked—diffuse marked with multifocal solid areas of inflammation devoid of glands; 5: severe—diffuse severe with large areas of mucosa devoid of glands. The parameters reflecting gland loss or surface erosion were scored individually based upon the percentage of total mucosa area affected: 0: none; 1: 1–10%; 2: 11–25%; 3: 26–50%; 4: 51–75%; 5: 76–100%.

### 2.6. Statistical Analysis

Data are presented as mean ± SEM and were analyzed using Graphpad Prism 4.03 (Graphpad Software, La Jolla, CA). Statistical analysis was performed using unpaired Student's *t*-tests versus relevant control or Log rank test, as appropriate.

## 3. Results

### 3.1. CCL25 Is Expressed in Normal Human Colon

**Figure 1:**
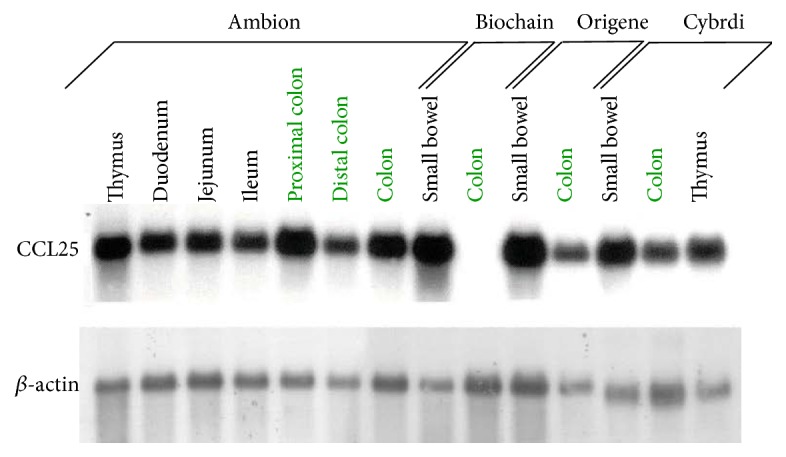
CCL25 mRNA is expressed in normal human colon. Normal intestinal mRNA samples purchased from multiple vendors were evaluated for CCL25 mRNA content by Northern Blotting. CCL25 mRNA was detected in 6 of 6 small intestine samples and 5 of 6 healthy colon samples.

**Figure 2:**
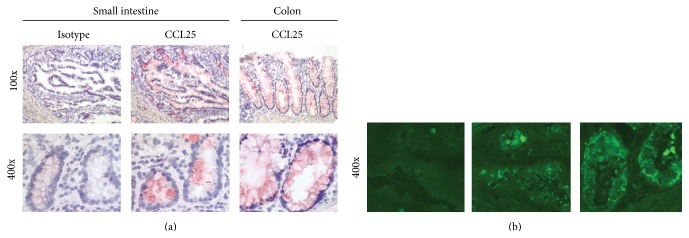
CCL25 protein is expressed in healthy human colon. Frozen sections of healthy human colon and small intestine were stained with CCL25 and isotype control antibodies. CCL25 protein was detected in epithelium in both colon and small bowel, as determined by IHC ((a), red color) and immunofluorescence ((b), green color). Magnification 100x (top row of (a)), 400x (bottom row of (a) and (b)).

Normal small intestine and colon mRNA samples from different vendors were evaluated for CCL25 transcripts by Northern blot analysis. CCL25 mRNA was detected in all small intestine samples and in the majority (5 of 6) of colon samples (Figure 1). Immunohistochemical and immunofluorescence methods were utilized to determine whether CCL25 protein is expressed in normal colon. By each method, CCL25 protein was detected on epithelial cells in both the small bowel and colon (Figure 2).

### 3.2. Colonic Inflammation Is Associated with Increased CCL25 Levels

**Figure 3:**
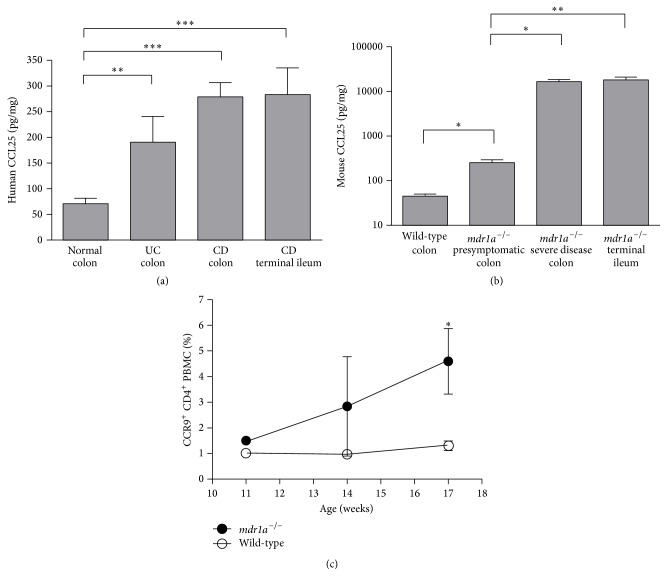
CCL25 protein levels are significantly elevated during colonic inflammation. CCL25 protein levels in homogenates of colon and terminal ileum were determined by ELISA and normalized to the levels of total protein. (a) CCL25 protein levels were significantly elevated in colon biopsies from individuals with active Crohn's disease or ulcerative colitis, relative to healthy controls. In Crohn's disease, the levels of CCL25 in the colon were equivalent to the levels in the terminal ileum. *n* = 6–8 individuals. (b) CCL25 levels were increased in presymptomatic* mdr1a* ^−/−^ mice and further increased in* mdr1a* ^−/−^ mice with severe disease, compared to wild-type mice. *n* = 6–8 mice. (c) The frequency of CCR9^+^CD4^+^ T cells among PBMC is significantly increased in* mdr1a* ^−/−^ mice compared to age-matched wild-type mice; *n* = 5 mice per time point. ^*∗*^ *P* < 0.05; ^*∗∗*^ *P* < 0.01.

Levels of CCL25 protein were measured in colon tissue from healthy individuals and Crohn's disease and ulcerative colitis patients, as well as Crohn's disease terminal ileum. CCL25 levels in colon biopsies from individuals with ulcerative colitis were significantly higher than CCL25 levels in normal colon (Figure 3(a)). CCL25 levels were also significantly higher in colon biopsies from individuals with active Crohn's disease (CDAI ≥ 250) (Figure 3(a)). Interestingly, CCL25 levels in the terminal ileum of Crohn's disease patients were not higher than the levels of CCL25 in the colon of these patients or ulcerative colitis patients (Figure 3(a)). As a specificity control, we found no CCL25 in psoriatic plaques (data not shown).

As* mdr1a* ^−/−^ mice spontaneously develop a form of colitis that exhibits many of the hallmarks of human ulcerative colitis, such as marked lymphocyte infiltration into the lamina propria, elevation of inflammatory cytokines, and ulceration [27, 28], we chose this model to examine the therapeutic benefit of pharmacological CCR9 inhibition in the context of colonic inflammation. This model is an attractive one, since it recapitulates the therapeutic activity of IL-12/23 p40 inhibition and the disease-exacerbating activity of IL-17 inhibition observed in humans [29]. In 16-week-old* mdr1a* ^−/−^ mice, which exhibited no overt symptoms (diarrhea, weight loss) of colitis, colonic CCL25 levels were significantly higher than in age-matched wild-type mice (Figure 3(b)). Colonic CCL25 levels in mice with severe colitis, defined by persistent, severe diarrhea and wasting, increased more than 60-fold (*P *< 0.01), compared to presymptomatic* mdr1a* ^−/−^ mice, and were more than 350-fold higher than in wild-type mice (*P *< 0.05; Figure 3(b)). CCL25 levels in the colon of* mdr1a* ^−/−^ mice with severe disease were similar to the levels of CCL25 in the small intestine of* mdr1a* ^−/−^ mice (Figure 3(b)). As a specificity control, we found no CCL25 in normal or inflamed brains from C57BL/6 mice with experimental autoimmune encephalitis (data not shown).

As a surrogate measure of intestinal inflammation we noted changes in the frequency of CCR9^+^ cells in the circulation of* mdr1a* ^−/−^ mice prior to the onset of clinical symptoms. The frequency of CCR9^+^CD4^+^ T cells in PBMC of wild-type mice remained ~1% over time (Figure 3(c)), whereas this frequency increased over time in* mdr1a* ^−/−^ mice. At 11 weeks of age, the frequency of CCR9^+^CD4^+^ T cells in* mdr1a* ^−/−^ mice was ~1.5% of PBMC, but this frequency was ~4.5% at 17 weeks of age (Figure 3(c)). No significant differences were noted between wild-type and* mdr1a* ^−/−^ mice with respect to the frequency of the CCR9^+^CD8^+^ peripheral T cell population (data not shown).

### 3.3. CCR9 Inhibition Confers a Significant Therapeutic Benefit in the* mdr1a* ^−/−^ Mouse Model of Ulcerative Colitis

The role of CCR9 in the initiation and progression of colitis was tested by pharmacological CCR9 inhibition with two small molecule antagonists, CCX282-B and CCX025. Both compounds are potent and selective antagonists of human and mouse CCR9 [12, 24]. CCX025 inhibits CCR9-mediated chemotaxis of human Molt-4 cells (a T cell line) with an IC_50_ value of 23 nM in buffer and 78 nM in 100% human serum (Supplementary Figures  S1A-B in Supplementary Material available online at http://dx.doi.org/10.1155/2015/628340). In addition, CCX025 inhibits CCR9-mediated chemotaxis of mouse thymocytes with an IC_50_ value of 12 nM in buffer (Supplementary Figure S1C). The IC_50_ values of CCX282-B have already been described [12]. For* in vivo *studies, the dose of CCX025 was selected based upon its ability to block T cell trafficking to the intestine (Supplementary Figure S1D), similar to the CCR9 antagonist CCX8037 [14]. The dose of CCX282-B was selected based on its ability to reduce ileitis severity in the TNF-ΔARE model [12].

**Figure 4:**
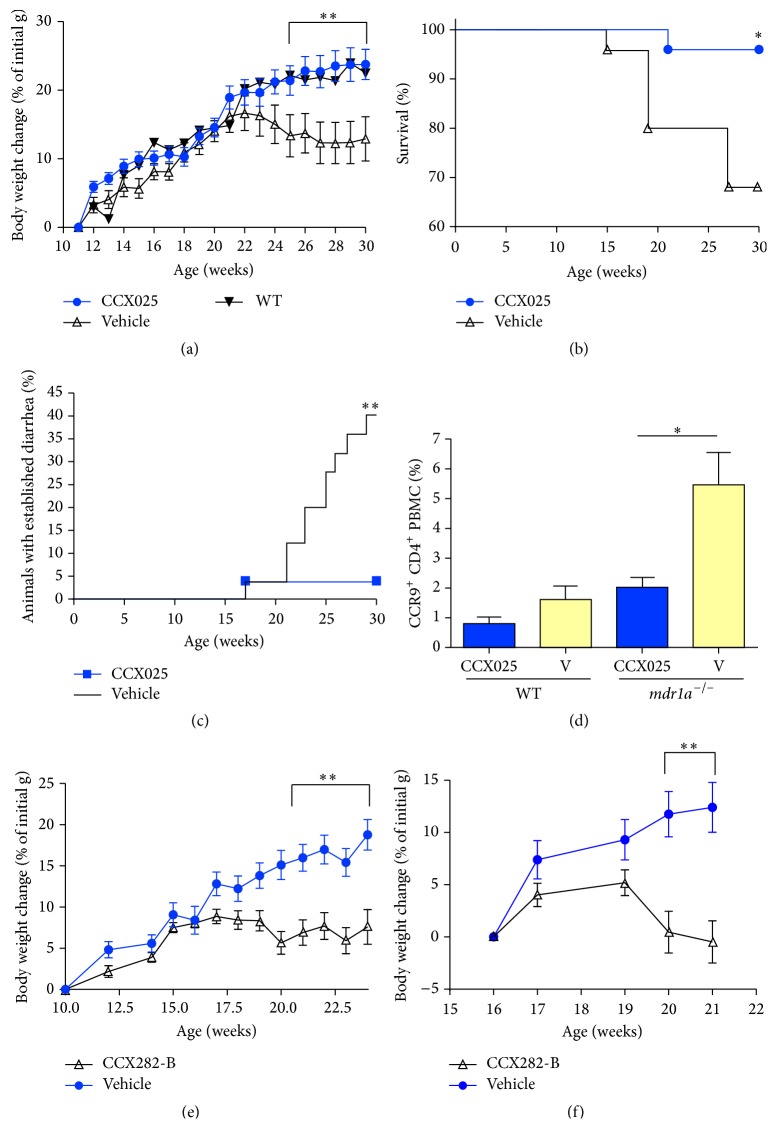
Pharmacological CCR9 inhibition results in a significant reduction in severity of colitis symptoms in* mdr1* ^−/−^ mice. (a) CCX025 treatment, starting at 11 weeks of age, resulted in a significant inhibition of the growth retardation associated with disease. Mice treated with CCX025 (*n* = 25) gained weight in an identical fashion to wild-type controls (*n* = 5); vehicle-treated* mdr1a* ^−/−^ mice (*n* = 25) failed to gain weight after week 22. (b) CCX025 treatment resulted in a significant reduction in mortality compared to vehicle in* mdr1a* ^−/−^ mice. (c) CCX025 treatment resulted in a significant reduction in the percentage of* mdr1a* ^−/−^ mice that developed established diarrhea (score of ≥3 out of 5). (d) CCX025 treatment resulted in a significant inhibition of the disease-induced increase in CCR9^+^CD4^+^ PBMC frequencies in* mdr1a* ^−/−^ mice, measured at 17 weeks of age. (e) CCX282-B treatment (*n* = 34), starting at 10 weeks of age, resulted in a significant inhibition of the growth retardation associated with disease, compared to controls (*n* = 29). (f) Therapeutic treatment with CCX282-B (*n* = 17 per treatment group) resulted in a significant reduction in the weight loss associated with active disease. Animals were randomized at 16 weeks of age to either CCX282-B or vehicle treatment. All panels: ^*∗*^ *P* < 0.05; ^*∗∗*^ *P* < 0.01.

*mdr1a* ^−/−^ mice were randomized into two groups and treated daily with either CCX025 or vehicle control, beginning at 11 weeks of age. Body weights and diarrhea incidence were recorded on a weekly basis. At 21 weeks of age, vehicle-treated* mdr1a* ^−/−^  mice began to exhibit weight loss consistent with the onset of colitis (Figure 4(a)). In contrast, CCX025-treated animals continued to gain weight at a rate similar to that of healthy wild-type mice, an effect that was maintained throughout the course of the study (Figure 4(a)). Because colitis-associated wasting is progressive,* mdr1a* ^−/−^ mice were euthanized if their body weight loss exceeded 20%. Over the course of the study, 32% of vehicle-treated* mdr1a* ^−/−^ mice had to be euthanized; in contrast, only 4% of CCX025-treated mice had to be euthanized (*P* < 0.05; Figure 4(b)). CCX025 also significantly reduced the incidence of established, severe diarrhea exhibited in this model (Figure 4(c)). At the end of the study, CCR9 expression on circulating T cells was assessed. Vehicle-treated* mdr1a* ^−/−^ mice exhibited a significant elevation in the frequency of CCR9^+^CD4^+^ T cells relative to wild-type mice; in contrast, CCX025-treated* mdr1a* ^−/−^ mice had normal levels of circulating CCR9^+^CD4^+^ T cells (Figure 4(d)).

CCX282-B was also tested in the* mdr1a* ^−/−^ model. Like CCX025, CCX282-B blocked the colitis-associated weight loss inherent in the model (Figure 4(e)). In order to test CCR9 inhibition in a nonprophylactic regimen, that is, starting treatment during the onset of colitis, 16-week-old* mdr1a* ^−/−^ mice were randomized to receive either CCX282-B or vehicle. Even when used in this regimen, CCX282-B abrogated growth arrest (Figure 4(f)). CCX282-B also caused a marked reduction in the incidence of established diarrhea (4% incidence with CCX282-B versus 24% with vehicle; data not shown).

**Figure 5:**
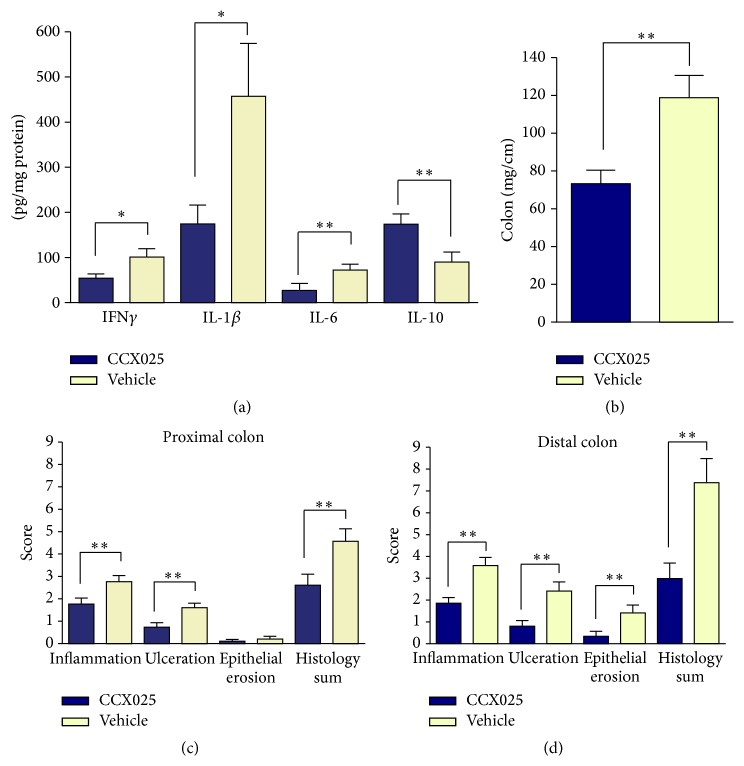
Pharmacological CCR9 inhibition results in a significant improvement in colonic inflammation in* mdr1a* ^−/−^ mice. (a) CCX025 treatment resulted in significantly lower levels of the proinflammatory cytokines IFN-*γ*, IL-1*β*, and IL-6 in the colon; at the same time CCX025 treatment resulted in a significant elevation in the levels of the anti-inflammatory cytokine IL-10 in the colon (*n* = 16–19). (b) CCX025 treatment, starting at 11 weeks of age, resulted in a significant reduction in the weight-to-length ratio of the colon in* mdr1a* ^−/−^ mice, compared to vehicle-treated mice (*n* = 25). CCX025 treatment resulted in significantly reduced histological damage scores in the proximal colon (c) and the distal colon (d). All panels: ^*∗*^ *P* < 0.05; ^*∗∗*^ *P* < 0.01.

We analyzed other important parameters of disease in CCX025-treated* mdr1a* ^−/−^ mice at the end of the study. First, colonic inflammation in the* mdr1a* ^−/−^ model is mediated in part by the proinflammatory cytokines IFN-*γ*, IL-1*β*, and IL-6. The levels of each of these cytokines were significantly reduced in the colons of* mdr1a* ^−/−^ mice treated with CCX025, relative to vehicle-treated mice (Figure 5(a)). In addition, CCX025 treatment was associated with a significant increase in the colonic levels of the anti-inflammatory cytokine IL-10, relative to vehicle-treated mice (Figure 5(a)). Second,* mdr1a* ^−/−^ mice exhibit structural changes in the colon, which become both shorter in length and heavier as a result of tissue remodeling [27, 28]. CCX025-treated mice exhibited a significant reduction in the colon weight : length ratio, relative to vehicle-treated mice (Figure 5(b)). Third,* mdr1a* ^−/−^ mice exhibit inflammation, ulceration, and epithelial erosion of the colon [27, 28]. CCX025 treatment resulted in a significant reduction in both the inflammation and ulceration scores in proximal colon of* mdr1a* ^−/−^ mice (Figure 5(c)). In the more severely affected distal colon, CCX025 treatment significantly reduced the inflammation, ulceration, and epithelial erosion scores (Figure 5(d)).

## 4. Discussion

In this study we show that the CCR9 ligand CCL25 is expressed by normal colon epithelium; that levels of colonic CCL25 increase in both Crohn's disease and ulcerative colitis; and that pharmacological inhibitors of CCR9 prevent the development of disease in an experimental model of ulcerative colitis. These results suggest that CCL25-mediated recruitment of CCR9^+^ leukocytes into the colon plays a causative role in the inflammation that occurs in chronic colonic diseases such as ulcerative colitis, similar to the case with ileal inflammation in Crohn's disease.

Several groups have failed to detect CCL25 in normal human colon [5, 9, 16], even though CCR9^+^ T cells are abundant there [5]. We used three methods to assess CCL25 expression in normal colon. Using Northern blotting on multiple samples from multiple vendors, we found that CCL25 mRNA is indeed expressed in human colon. Using ELISA, we found that CCL25 protein is expressed in both human and mouse colon. Finally, using IHC, we found that CCL25 protein is expressed by the colonic epithelium, as is the case in small intestine [5, 9, 18]. We surmise that the earlier studies' inability to detect CCL25 in normal colon was due either to insufficient amounts of epithelium in their samples or to the possibility that CCL25 is expressed unevenly over the length of the colon, as has been previously described for the small intestine [30].

Even though CCL25 protein levels in normal human and mouse colon were lower than in the small intestine, substantial elevations were observed in both species in connection with colonic inflammation. Colon biopsies obtained from patients with active Crohn's disease (CDAI ≥ 250) had significantly higher levels of CCL25 protein than healthy colon samples. The increase in colonic CCL25 levels was not restricted to Crohn's disease, since colon biopsies from ulcerative colitis patients also contained significantly elevated levels of CCL25 protein. In the* mdr1a* ^−/−^ mouse model of ulcerative colitis, increased colonic CCL25 protein levels were noted before any clinical manifestations of disease and correlated temporally with increases in the frequency of circulating CCR9^+^CD4^+^ T cells, which we used as a surrogate for intestinal inflammation. Colonic CCL25 protein levels continued to increase dramatically as mice developed severe colitis, and the highest levels of colonic CCL25 protein were found in those mice with the most severe disease. An increase in CD4^+^ cells in the colonic lamina propria lymphocyte and intraepithelial lymphocyte populations of* mdr1a* ^−/−^ mice with active colitis has been described [27, 28]; it may well be that such an increase results from the concurrent upregulation of CCL25 in the colon coupled with an increase in circulating CCR9^+^CD4^+^ T cells, both documented in our studies.

Interference with the CCR9/CCL25 axis has been shown to improve disease symptoms and tissue inflammation in mouse models of ileitis such as the SAMP-1/Yit model [13] and the TNF-ΔARE model [12]. The* mdr1a* ^−/−^ mouse model shares many features with human ulcerative colitis, such as lymphocyte infiltration into the lamina propria and epithelial destruction and ulceration, and thus is a robust model in which the efficacy of new therapeutic agents is to be tested [27, 28]. Treatment of* mdr1a* ^−/−^ mice with the CCR9 antagonist CCX025 resulted in complete protection from the development of colitis. All aspects of the disease were ameliorated, including weight loss, diarrhea, inflammation, colon shortening, and mortality. These effects were also noted with the CCR9 antagonist CCX282-B, indicating that the CCX025-mediated changes were due to CCR9 inhibition and not the result of an off-target effect. While our results are consistent with reports that describe inflammation throughout the entire colon [27], we observed, histologically, a higher degree of inflammation in the distal colon compared to the proximal colon.

CCR9 inhibition resulted in improvements in the degree of ulceration and epithelial erosion in the colon. It is reasonable to assume a causal relationship between decreased lymphocyte infiltration, histological improvements, and improved clinical symptoms. The importance of mucosal healing to the clinical manifestation of ulcerative colitis has been described [31]. Inhibition of CCR9 also resulted in marked changes in the cytokine profiles observed in colonic tissue from* mdr1a* ^−/−^ mice. Significant decreases were seen in the levels of IFN-*γ*, IL-1*β*, and IL-6, all of which are elevated in human lamina propria mononuclear cells isolated from ulcerative colitis and Crohn's disease patients [32]. The reduced levels of IFN-*γ* are noteworthy, as CCR9^+^ cells have been shown to be a significant source of this cytokine in Crohn's disease [33]. The mice treated with CCX025 also had increased colonic levels of the anti-inflammatory cytokine IL-10, in concordance with the fact that IL-10^−/−^ mice have a predisposition to develop colonic inflammation [34]. Finally, CCR9 inhibition resulted in normalization of the frequency of circulating of CCR9^+^CD4^+^ PBMC.

The results presented herein clearly demonstrate that pharmacological inhibition of CCR9 provides a therapeutic benefit in the* mdr1a* ^−/−^ mouse colitis model. However, there are reports in the literature that conclude that CCR9^−/−^ mice fare worse in the DSS-induced acute colitis model [19] and are unable to develop tolerance to oral antigens [35]. Differences between CCR9 pharmacological inhibition and genetic deletion have also been reported in the TNF-ΔARE ileitis model: pharmacological inhibition of CCR9 [12] was beneficial, whereas CCR9 deletion either had no effect [36] or worsened disease [37]. We recently conducted a head-to-head comparison between the effects of CCR9 pharmacological inhibition and genetic deletion on oral tolerance in mice and found that CCR9 deletion does not predict the results obtained when using a pharmacological approach [38]. The inability of the CCR9^−/−^ mouse to predict the outcome of pharmacological inhibition could be explained by differences in the intestinal composition of immune cells (such as *γδ* T cells [39], plasmacytoid DC [7], and IgA^+^ plasma cells [40]) reported in CCR9^−/−^ mice relative to wild-type mice.

One limitation of our study is that we have not proven the mechanism of action of the CCR9 antagonists in the* mdr1a* ^−/−^ model. We surmise the mechanism to be the inhibition of CCR9^+^ T cell trafficking to the colon, based on short-term trafficking models in which CCR9 antagonists blocked antigen-mediated T cell trafficking to the small intestine of OT-1 mice [14] (and Figure S1D). Another limitation lies in the fact that activities occurring in mouse models of disease do not always recapitulate in human patients. However, given the lack of safe, orally administered treatments for ulcerative colitis, the increase in colonic CCL25 levels (Figure 3) and circulating CCR9^+^ leukocytes [17, 20] during disease, and the remarkable activities of CCR9 antagonists in the* mdr1a* ^−/−^ model of ulcerative colitis (Figures 4 and 5), exploring CCR9 antagonism as a therapy for ulcerative colitis seems appropriate. CCX282 and CCX025 have been shown to be safe and well-tolerated in healthy subjects in Phase I clinical trials, and trials in patients with colonic inflammatory disorders such as ulcerative colitis are indicated.

## Supplementary Material

CCX025 is a potent inhibitor of T-cell migration. Increasing concentrations of CCX025 inhibit the chemotaxis of Molt-4 T-cells which constitutively express human CCR9, to human CCL25 in an *in vitro* migration assay. CCX025 was potent when the assay was run in buffer (a) or in 100% human serum (b). CCX025 inhibits the *in vitro* chemotaxis of primary mouse thymocytes to murine CCL25 (c). In an *in vivo* T-cell trafficking model in mice, CCX025 inhibited the recruitment of CD8+ T-cells to the small intestine of OT-1 mice given an oral challenge of ovalbumin (d)
