# Supplementary material for: CCR9 Antagonists in the Treatment of Ulcerative Colitis
Source: Mediators Inflamm. 2015 Sep 20;2015:628340. doi: 10.1155/2015/628340 (PMC4592714; doi:10.1155/2015/628340)
Supplement: Supplementary file 1 — CCX025 is a potent inhibitor of T-cell migration. Increasing concentrations of CCX025 inhibit the chemotaxis of Molt-4 T-cells which constitutively express human CCR9, to human CCL25 in an in vitro migration assay. CCX025 was potent when the assay was run in buffer (a) or in 100% human serum (b). CCX025 inhibits the in vitro chemotaxis of primary mouse thymocytes to murine CCL25 (c). In an in vivo T-cell trafficking model in mice, CCX025 inhibited the recruitment of CD8+ T-cells to the small intestine of OT-1 mice given an oral challenge of ovalbumin (d) [file 628340.f1.pdf]

Supplementary Materials:

Fig. S1. CCX025 is a potent inhibitor of CCR9-mediated cell migration in vitro and in vivo.

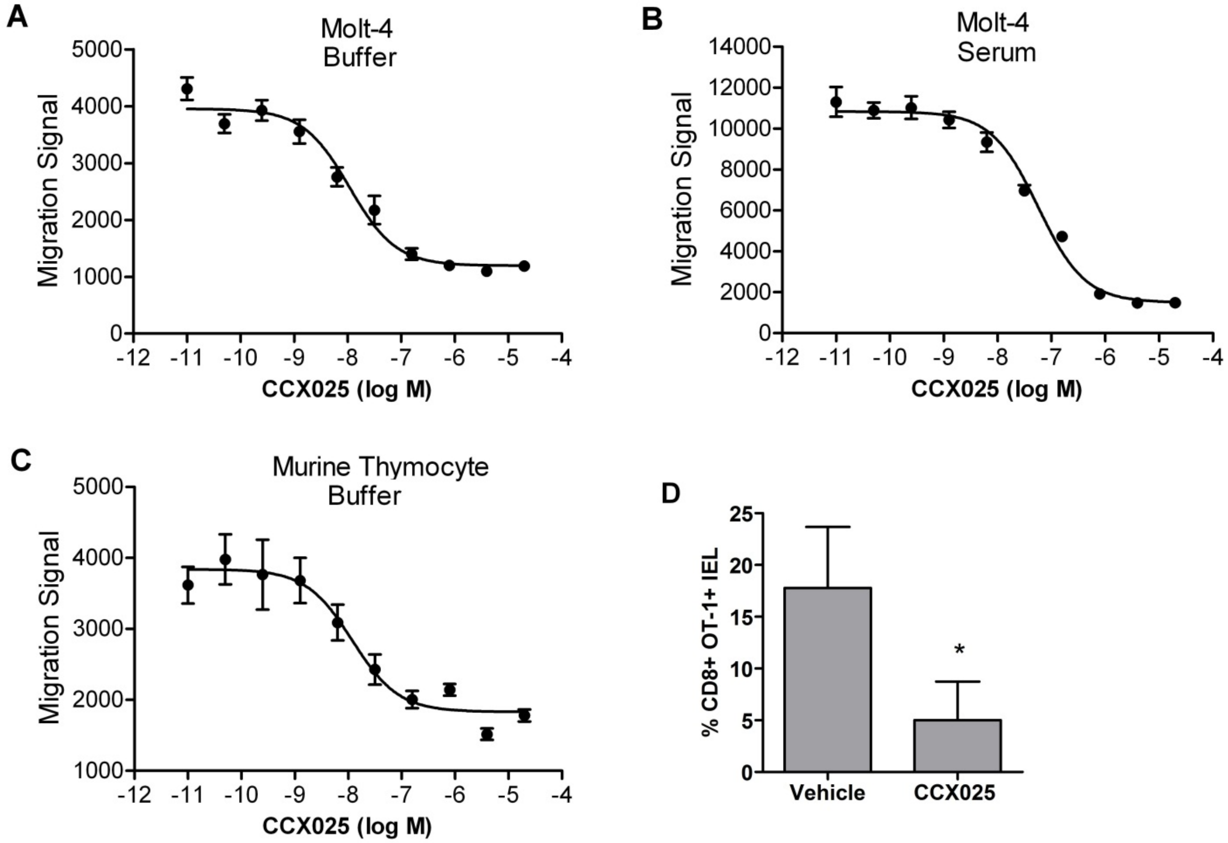

Fig. S1. CCX025 is a potent inhibitor of CCR9-mediated cell migration in vitro and in vivo. (**A-B**): CCX025 blocks the chemotaxis of human Molt-4 cells to 80 nM CCL25 in buffer (**A**) and 100% human serum (**B**) with IC_{50} values of 23 nM (n=9) and 78 nM (n=6), respectively. (**C**): CCX025 blocks the chemotaxis of mouse thymocytes to 80 nM CCL25 in buffer with an IC_{50} value of 12 nM. (**D**): CCX025 blocks the migration of adoptively-transferred ovalbumin-specific OT-1 T $CD8^+$ T cells to the small intestine after oral gavage with ovalbumin.
